# Comparative Analysis of Single-Cell Transcriptome Data Reveals a Novel Role of Keratinocyte-Derived IL-23 in Psoriasis

**DOI:** 10.3389/fimmu.2022.905239

**Published:** 2022-05-25

**Authors:** Young Joon Park, Yul Hee Kim, Eun-So Lee, You Chan Kim

**Affiliations:** Department of Dermatology, Ajou University School of Medicine, Suwon, South Korea

**Keywords:** psoriasis, keratinocyte-derived IL-23, single-cell RNA sequencing, IL-23, IL-36

## Abstract

Psoriasis, a common inflammatory skin disease, is critically dependent on the IL-23/IL-17 cytokine axis. Although immune cell-derived IL-23 is generally associated with the disease pathogenesis, there have been reports of IL-23 production in keratinocytes. To determine the presence and potential role of keratinocyte-derived IL-23 in psoriasis, we investigated its expression levels using publicly available single-cell RNA sequencing data from human samples. We discovered that the expression of IL23A was detectable in keratinocytes as well as dendritic cells. Furthermore, we examined the IL-23p19 expression in an imiquimod-induced mouse model of psoriasis and found a close relationship between keratinocyte-produced IL-23 and IL-36, another key cytokine in psoriasis pathogenesis. The blockade of IL-23 signaling resulted in the reduced expression of IL-36 in the keratinocytes. Our findings reveal the novel association between keratinocyte-derived IL-23 and IL-36 in psoriasis progression.

## Introduction

Psoriasis is an inflammatory skin disease that lowers the quality of life and daily function of an individual and may lead to multiple comorbidities, such as psoriatic arthritis and coronary artery disease, when not properly treated. The classical clinical morphology of psoriasis is characterized by well-demarcated, erythematous plaques with scales, while the characteristic histopathologic findings include epidermal thickening with downward elongation of rete ridges, immune cell infiltration into the dermis and epidermis, and neutrophil clusters (1). Recent studies on the pathogenesis of psoriasis have shown that psoriatic inflammation is critically dependent on the IL-23-induced production of IL-17 by lymphocytes, especially T helper cells (2). The improved understanding of psoriasis has resulted in the development of biological drugs targeting the IL-23/IL-17 immune axis (3). For example, multiple IL-23 inhibitors demonstrated high efficacy and safety for treating plaque psoriasis, thereby highlighting the importance of cells producing IL-23, specifically myeloid cells (i.e., macrophages, monocytes, and dendritic cells) (4–6). Interestingly, keratinocytes also express the mRNA for the p19 and p40 subunits of IL-23, which was found to have higher expression in psoriasis lesional skin compared to non-lesional skin (7). A recent study also reported that keratinocyte-derived IL-23 can cause epidermal thickening and cellular infiltration resembling psoriasis symptoms in mice (8). Furthermore, the continuous elevated expression of IL-23 in keratinocytes showed similar symptoms of psoriatic arthritis, such as enthesitis, dactylitis, and bone destruction (9). Therefore, we aimed to re-analyze previously published single-cell RNA sequencing (scRNA-seq) datasets to confirm IL-23 production in keratinocytes and to investigate the possible roles of keratinocyte-derived IL-23 in psoriasis.

## Results

### Psoriatic Keratinocytes Possess Significant Levels of *IL23A*

**Figure 1:**
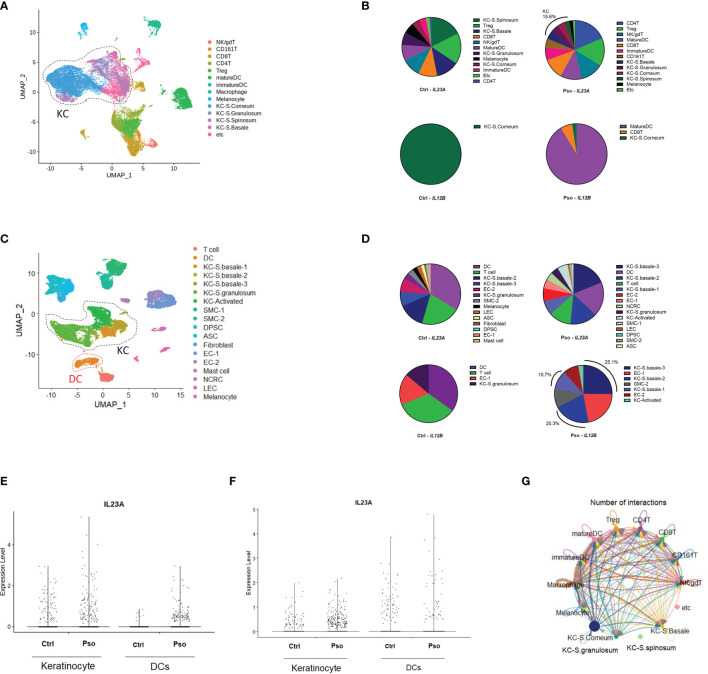
Keratinocyte-derived *IL23A* expression in the GSE151177 and GSE162183 datasets. **(A)** Uniform Manifold Approximation and Projection (UMAP) of the single-cell RNA sequencing (scRNA-seq) data from GSE151177. **(B)** Percentage of each IL23A-expressing cluster in GSE151177. **(C)** UMAP of the scRNA-seq data from GSE162183. **(D)** Percentage of each IL23A-expressing cluster in GSE62183. **(E, F)** *IL23A* expression levels in the DCs and keratinocytes of GSE151177 **(E)** and GSE162183 **(F)**. **(G)** CellChat network showing the number of interactions between clusters in GSE151177.

We downloaded the scRNA-seq datasets GSE151177 and GSE162183 from the Gene Expression Omnibus (GEO) database and used the Seurat platform workflow for bioinformatics analysis, following the methods in the original articles (10–12). Using the FeaturePlot function, DotPlot function, and differentially expressed gene (DEG) markers among clusters, we identified 14 and 18 cell lineages from the GSE151177 and GSE162183 datasets, respectively (**Figures 1A, C**; **Supplementary Figures S1A, B**). We counted the cells with *IL23A* and *IL12B* expression higher than 0 (expression level >0) and calculated the proportion of *IL23A*-expressing cells in each cell cluster. We observed that there was a substantial proportion of *IL23A*-expressing keratinocytes regardless of the patient group, i.e., healthy control and psoriasis patients (**Figures 1B, D**). In psoriasis skin lesions, keratinocytes were the second most prevalent source of *IL23A* in GSE151177 and the primary source of *IL23A* in GSE162183. In contrast, despite the very low numbers of *IL12B*-expressing cells, a substantial proportion of *IL12B* was expressed in psoriatic keratinocytes and healthy controls (**Figures 1B, D**). Due to the importance of IL-23 production in dendritic cells (DCs), we grouped the keratinocyte clusters (KC-S.corneum, KC-S.granulosum, KC-S.spinosum, and KC-S.basale for GSE151177; KC-S.basale-1, KC-S.basale-2, KC-S.basale-3, KC-S.granulosum, and KC-Activated for GSE162183) and DC clusters (immature and mature DC for GSE151177; DC for GSE162183) for the comparison of *IL23A* expression. As expected, the elevated *IL23A* expression in the psoriatic DCs was detected in both datasets. Interestingly, compared to healthy controls, a similar increase in *IL23A* expression was observed in psoriatic keratinocytes (**Figures 1E, F**). In GSE162183, the *IL23A* expression levels were higher in DCs than in keratinocytes (**Figure 1F**). Thus, we hypothesized that only a small amount of IL-23 is secreted from keratinocytes, likely influencing the surrounding keratinocytes and not the infiltrating immune cells. We also analyzed the number of interactions using the CellChat package and discovered substantial interactions between keratinocytes from different skin layers (**Figure 1G**; **Supplementary Figure S1C**).

### IL-23 Production in the Keratinocytes of Imiquimod-Treated Mice

**Figure 2:**
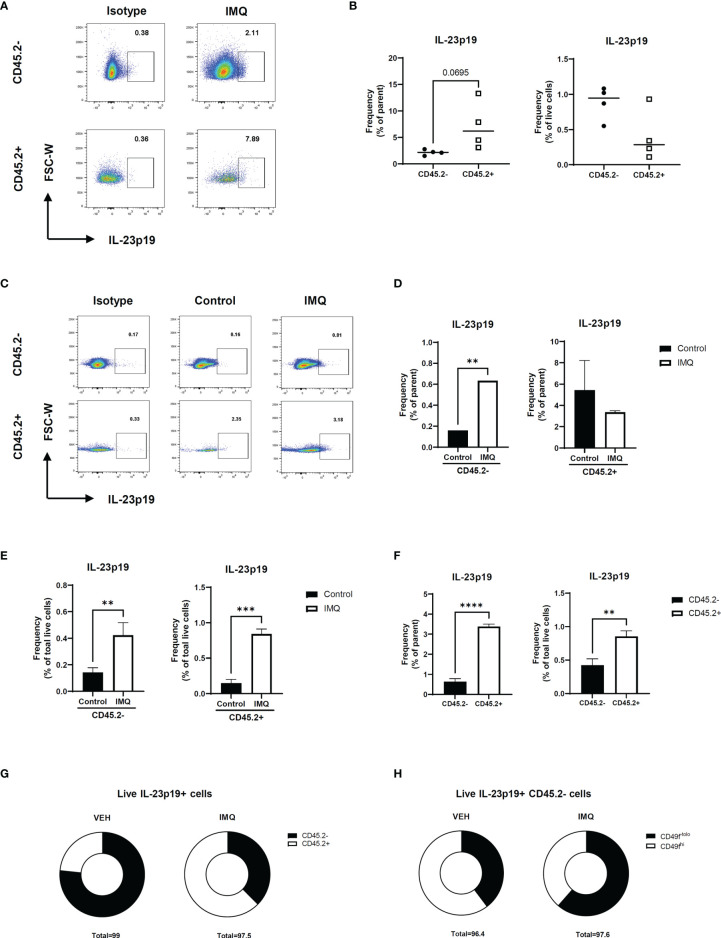
IL-23 production in the keratinocytes of imiquimod-treated mice. **(A, B)** Representative flow cytometry plot **(A)** and graph **(B)** showing the frequency of IL-23p19+ cells in imiquimod-treated mice skin. **(C–E)** Representative flow cytometry plot **(C)** and graphs **(D, E)** showing the frequency of IL-23p19+ cells in imiquimod-treated mice epidermis. **(F, G)** Bar graphs **(F)** and pie charts **(G)** showing the proportion of IL-23p19+ cells in CD45.2- and CD45.2+ cells of the epidermis. **(H)** Percentage of CD49^-tolo^ cells and Cd49^hi^ cells in live IL-23p19+ Cd45.2^-^ cells. Data are expressed as means ± SD. Lines above bars indicate statistical comparisons with significant differences between categories (**P < 0.01; ***P < 0.001; ****,*P*< 0.0001). Data represent two independent experiments.

Prior to investigating the effect of IL-23 in other keratinocytes, we investigated IL-23 production in the keratinocytes using a mouse model of psoriasis. The intracellular staining of the IL-23p19 subunit and its isotype revealed an evident production of IL-23p19 in the CD45.2^-^ and CD45.2+ populations (**Figures 2A, B** and **Supplementary Figure S2A**). To confirm whether the keratinocytes of CD45.2^-^ populations are capable of IL-23 production, we divided the ear halves of the control and imiquimod-treated mice (which showed an evident increase in ear thickness; **Figure S2B**) into the epidermis and dermis and analyzed the epidermis using flow cytometry (**Supplementary Figure S2C**). The percentage of IL-23p19+ CD45.2+ cells increased in the epidermis after imiquimod treatment as expected, and the epidermal CD45.2^-^ cells, which are most likely keratinocytes, also showed increased levels of IL-23p19 upon treatment (**Figures 2C–E**). Interestingly, despite the significant difference between the frequency of IL-23p19+ CD45.2^-^ and IL-23p19+ CD45.2+ cells in the epidermis (**Figure 2F**), a substantial percentage of CD45.2^-^ cells produced IL-23p19 on imiquimod treatment (**Figure 2G**). Since basal keratinocytes express integrin α6 (CD49f) (8), we compared the CD49f expression in IL-23p19+ CD45.2^-^ cells and discovered that the IL-23p19 increase upon imiquimod treatment was due to suprabasal keratinocytes, as evidenced by the decreased CD49f^hi^ proportion in IL-23p19+ CD45.2^-^ cells (**Figure 2H**). Thus, we speculated that adjacent suprabasal keratinocytes may be directly affected by keratinocyte-derived IL-23.

### IL-36 Expression Is Elevated in Suprabasal Keratinocytes and Influenced by IL-23 in Psoriasis

**Figure 3:**
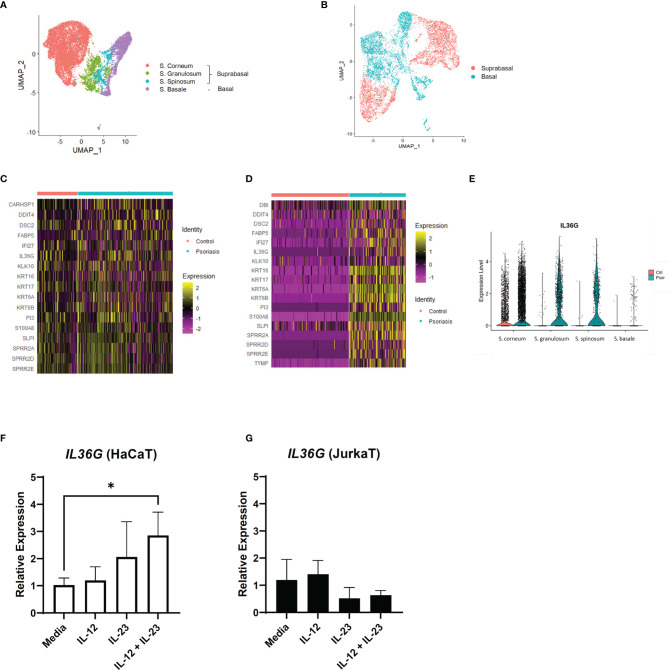
*IL36G* and IL-23 expression in the keratinocytes of psoriasis patients and healthy controls. **(A)** Uniform Manifold Approximation and Projection (UMAP) of the keratinocyte cluster in GSE151177. **(B)** UMAP of the GSE162183 keratinocyte cluster based on *KRT* expression. **(C, D)** Differentially expressed genes between the healthy and psoriatic suprabasal epidermis in the keratinocyte clusters of GSE151177 **(C)** and GSE162183 **(D)**. Features omitted in the heatmap are due to the lack of data in the scale data slot of the studied datasets. **(E)** Violin plot of the *IL36G* expression in the different epidermal layers (GSE151177). **(F, G)** Relative expression of *IL36G* in the HaCaT cells after stimulation by recombinant human IL-12 and/or IL-23. Data are expressed as means ± SD. Lines above bars indicate statistical comparisons with significant differences between categories (*P < 0.05).

We extracted the keratinocyte group from the previous scRNA-seq data for a detailed analysis. The keratinocytes in GSE151177 were differentiated into different layers based on the following markers: *FABP5*, *KRT1*, *KRT5*, *KRT10*, *KRT14*, and *SPRR2G* (**Figure 3A** and **Supplementary Figures 3A, B**). In GSE162183, the layers of keratinocytes were not fully differentiated using the same markers because there was only a low number of *CDSN*- and *SPRR2G*-expressing cells (**Supplementary Figure S3C**) (10). Notably, the suprabasal layer was successfully differentiated from the basal layer using *KRT1* and *KRT5* (**Figure 3B**; **Supplementary Figure S3D**). Therefore, we grouped S. corneum, S. granulosum, and S. spinosum in GSE151177 as the suprabasal layer (**Figure 3A**) and compared the DEGs in the suprabasal keratinocytes between the diseased and healthy states. The top 25 most common DEGs in the suprabasal keratinocytes between GSE151177 and GSE162183 both included *IL36G*, a keratinocyte-derived cytokine known as an important factor in psoriasis pathogenesis (**Figures 3C, D**). The increased presence of IL-36 in was mainly seen in the suprabasal layer of psoriatic epidermis (**Figure 3E**, **Supplementary Figure 3E**) As strong IL-23R expression is detected in psoriatic epidermis (13), we presumed that IL-23 might be associated with IL-36 production. We used HaCaT and JurkaT cell lines to check the possible association between IL-23 and IL-36 γ. We stimulated the cell lines with IL-12 and IL-23 and discovered that IL-23 expression, especially in conjunction with IL-12, induced *IL36G* expression in the keratinocytes but not in the T cells (**Figures 3F**, **G**; **Supplementary Figure 3F**).

### Systemic Treatment Using Anti-IL12/23 p40 Antibody Reduces IL-36γ Expression in Keratinocytes

**Figure 4:**
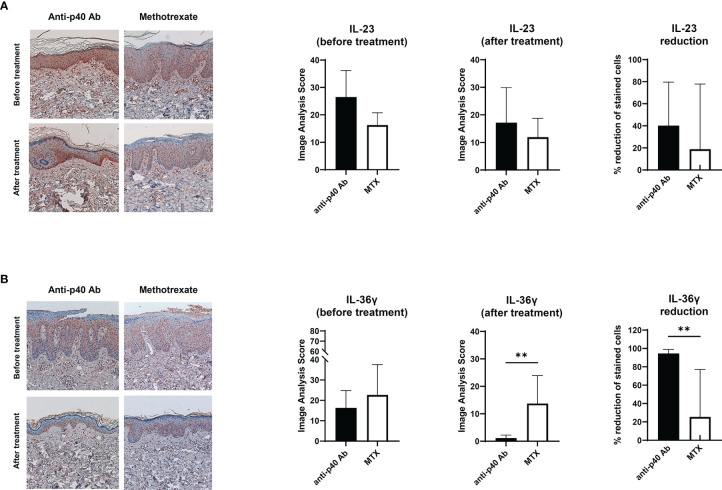
Immunohistochemical analysis of the IL-23 and IL-36 expression in psoriatic epidermis. **(A)** IL-23 expression on paraffin-embedded sections of biopsies obtained from psoriatic skin treated with anti-p40 monoclonal antibody (ustekinumab) (n=8) and methotrexate-treated skin (n=9). **(B)** IL-36γ expression on paraffin-embedded sections of biopsies obtained from psoriatic skin treated with anti-p40 monoclonal antibody (ustekinumab) (n=8) and methotrexate-treated skin (n=9). Lines above bars indicate statistical comparisons with significant differences between categories (**P < 0.01). Data represent two independent experiments.

To confirm the relationship between IL-23 and IL-36γ *in vivo*, we retrospectively reviewed the skin biopsies obtained from psoriasis patients who underwent anti-IL12/23 p40 monoclonal antibody (ustekinumab, Stelara^®^) treatment and compared them with psoriasis patients who received oral methotrexate therapy. We hypothesized that as ustekinumab blocks IL-12 and IL-23, there would be more changes in epidermal IL-36γ expression when compared with methotrexate. The demographics and psoriasis area and severity index (PASI) scores of the two groups are shown in **Table 1**. There were no statistically significant differences between the two groups. We also compared the histopathologic changes between the drug-treated and oral-medicated groups (**Table 2**). The ustekinumab-treated group showed a greater reduction in regular acanthosis (p=0.008), clubbed and elongated rete ridges (p=0.01), and perivascular inflammatory cell infiltration (p=0.027) than the methotrexate-treated group, suggesting that ustekinumab caused a more significant reduction in psoriatic plaques. Results of histopathologic analysis suggest that despite the insignificant difference in PASI, there was a significant repression of psoriasis pathogenesis by ustekinumab compared with methotrexate. To observe cytokine changes in the epidermis, we performed immunohistochemical staining using various cytokines, including IL-23, IL-36γ, IL-17A, and IFN-γ. The epidermal image analysis score (the ratio of the stained epidermal area to the measured epidermal area) was compared between the two groups. Despite significant lower IL-23 expression in the epidermis of the ustekinumab-treated group, there was no statistically significant difference between the two groups (**Figures 4A, B**). There was a significant difference between the image analysis score of IL-36γ in ustekinumab- and methotrexate-treated keratinocytes (**Figures 4C, D**). The IL-17A and IFN-γ levels were not significantly different between the two groups (**Supplementary Figures S4A, B**). Overall, the systemic blockade of IL12/IL23 signaling resulted in the reduced expression of IL-36γ in the keratinocytes, thereby suggesting the direct effect of IL-23 on IL-36γ production in psoriatic keratinocytes.

**Table 1:** Demographics of ustekinumab- and methotrexate-treated psoriasis patients.

Demographics			
	Ustekinumab	Methotrexate	*P*
Case No.	8	9	
Age, median [IQR], years old	37.0 [31.0; 42.8]	42 [38.5; 55.0]	0.2
Disease duration, median [IQR], years	7.6 [6.3; 10.9]	2.8 [0.6; 20.0]	0.606
Sex, N (%)			0.541
Male	6 (75.0)	5 (55.5)	
Female	2 (25.0)	4 (44.5)	
Treatment duration, median [IQR], weeks	100 [73; 175]	43 [12; 113]	0.114
PASI score before treatment, median [IQR]	12.4 [10.9; 14.9]	11.3 [6.6; 16.9]	0.541
PASI score after treatment, median [IQR]	3 [2.1; 9.7]	10 [5.3; 10.9]	0.2

IQR, interquartile range.

**Table 2:** Histopathologic features of ustekinumab- (biologics) and methotrexate-treated psoriasis (oral medication) patients.

Histological features					
Histopathologic features	Ustekinumab		Methotrexate		*P*
	baseline (n = 8)	after treatment (n =8)	baseline (n = 9)	after treatment (n =9)	
Parakeratosis					0.167
Scant	1	6	2	3	
Prominent	7	2	7	6	
Regular acanthosis	6	3	7	8	0.008
Munro’s microabscess	3	2	6	3	0.541
Spongioform micropustules of Kogoj	4	1	1	3	0.088
Epidermal spongiosis					0.541
Slight	0	6	2	6	
Prominent	8	2	7	3	
Clubbed rete ridges	7	2	7	7	0.010
Thinning of suprapapillary plates	6	1	6	4	0.243
Dermal inflammatory cell infiltration					0.027
Mild	3	6	1	1	
Moderate	4	2	5	7	
Dense	1	0	3	1	

## Discussion

In this study, we investigated the IL-23 production in keratinocytes and the potential role of keratinocyte-produced IL-23 in psoriasis using multiple modalities, including scRNA-seq analysis, experiments using an animal model and two cell lines, and immunohistochemistry. Several studies have investigated the possibility of keratinocyte-producing IL-23 (7–9). No study, however, has investigated keratinocyte-derived IL-23 using scRNA-seq. Hence, we used two scRNA-seq datasets to analyze IL-23 production and confirmed *IL23A* expression in the keratinocytes. Despite the differences in the methods for isolating the cells and the treatments before library preparation (10, 12), a substantial proportion of *IL23A* was produced by keratinocytes in both datasets, thereby validating the production of IL-23. IL-23 from myeloid cells stimulates T cells to produce IL-17 (14, 15), a major pathogenic cytokine in psoriasis. However, IL-23, especially those from suprabasal keratinocytes, might also play other roles in psoriasis, as these cells are located distant from the dermis, where T cell infiltration occurs. Thus, we analyzed the changes in the keratinocytes in psoriasis and identified keratinocyte-derived IL-23 as a novel stimulus of IL-36γ production.

IL-36 has been associated with various diseases, especially systemic inflammatory diseases (16). The relationship between IL-36 and generalized pustular psoriasis has been confirmed (17, 18). With the success of anti-IL-36 receptor monoclonal antibody treatment in generalized pustular psoriasis (19), the potential role of IL-36 in the pathogenesis of chronic plaque-type psoriasis has also been reported (19, 20). Furthermore, IL-36 is known as an upstream signaling molecule of IL-23 (21). Our findings suggest that IL-23 is not only a product of upstream IL-36 signaling, but also the stimulus for keratinocytes to produce IL-36. As IL-23 affects IL-36 production and vice versa, the cytokines could form an auto-inflammatory loop, amplifying the initial response to a pathogenic state. The auto-inflammatory loop might partly explain the Koebner phenomenon in psoriasis, in which the lesion appears at the site of repetitive trauma. A previous study reported the increased IL-23 production in the epidermis after only a mild tape-stripped injury (22). Trauma may lead to IL-23 increase in the epidermis which would lead to increase in IL-36, thereby initiating the auto-inflammatory loop.

The limitation of our study is that we did not identify mediators to confirm the direct effect of IL-23 on IL-36 production. A topical agent that effectively inhibits epidermal IL-23 production may also show a significant effect on IL-36 and would help delineate the mechanism of IL-36 production; however, this type of drug is currently unavailable. Further studies will be required to explore the potential mediators between IL-12 and IL-36. In conclusion, our findings provide evidence on the presence of keratinocyte-produced IL-23 in psoriasis and suggest that it might affect IL-36 production. Future research on the local inhibition of IL-23 production in keratinocytes may be a potential treatment strategy for plaque psoriasis.

## Materials and Methods

### Data and Code Availability

The raw scRNA-seq datasets (GSE151177 and GSE 162183) were obtained from the GEO database. The GSE151177 dataset was generated using human skin samples obtained from 13 psoriasis patients and five healthy volunteers, while the GSE162183 dataset was obtained from three psoriasis patients and healthy controls each. The detailed demographics of the subjects were reported in previously published papers (10, 12). The code details (scripts) used in this study are available from the authors upon request.

### ScRNA-Seq Data Analysis

The scRNA-seq data analysis was performed using Seurat R package (version 4.0.5) (11). Prior to normalizing gene expression, we discarded the data from cells that expressed <100 genes and those that expressed >25% of mitochondrial genes. Variable genes from cells were identified using the FindVariableFeatures function, following the vst method. The samples were scaled, and principal component analysis was conducted. The datasets for each group were integrated using the FindIntegrationAnchors and IntegrateData functions for GSE151177. The cells were then clustered using the FindClusters function, with 0.8 and 0.25 resolution for GSE151177 and GSE162183, respectively. The generated data were visualized using Uniform Manifold Approximation and Projection (UMAP) with a dimension of 1:20.

### Cell Type Annotation, Visualization of Gene Expression, and Cell-to-Cell Communication

To identify the cell-type clusters, the DEGs in each cluster were gathered using the FindMarkers function and subsequently filtered using Bonferroni method with adjusted p-value <0.05. The cell types were annotated based on the DEGs (**Supplementary Figures S1A, B**). To compare the gene expression in each cell type or group, DotPlot, FeaturePlot and VlnPlot were used. In addition, the gene expression data for each cell was collected and compared between cells. The CellChat R package was used to visualize the interaction between cells (23).

### Mice

The C57BL/6J male mice were obtained from Jackson Laboratory (Bar Harbor, ME, USA) and bred in-house in an animal facility at the Ajou University School of Medicine. The housing, breeding, and experimental procedures involving animal subjects were performed in accordance with the institutional guidelines. The study design was approved by the Committee for Ethics in Animal Experiments of the Ajou University School of Medicine.

### Induction of Psoriasis-Like Skin Inflammation

Mice aged 8 to 12 weeks were used to establish the psoriasis model. Twenty milligrams of 5% imiquimod cream (Aldara^®^; 3M Pharmaceuticals, Leicestershire, UK) was applied daily to the skin of both ears for five consecutive days. The mice were sacrificed on day 6 for subsequent analysis. Ear thickness was measured daily using a digital micrometer (293-821-30; Mitutoyo Corporation, Kawasaki, Japan).

### Single-Cell Preparation From Skin

Whole skin cells were prepared from the mouse ear skin. The cartilage of the ear was removed, and the dorsal and ventral pieces were separated. The ear halves were incubated in 2 mg/mL Dispase^®^ II (Roche, Mannheim, Germany) for 1 h at 37°C. The dermis was scraped off the ear halves. Both epidermis and dermis were cut into small pieces and digested with DMEM containing 1% FBS, 1 mg/mL collagenase IV (Worthington Biochemical Corp., Lakewood, NJ, USA), 0.1 mg/mL DNase I (Roche), and 1.2 mg/mL hyaluronidase (Sigma-Aldrich, St. Louis, MO, USA) for 30 min at 37°C. The digested skin tissues were plunged through 70-µm cell strainers. The cells were treated with ACK Lysis Buffer (Thermo Fisher Scientific, Waltham, MA, USA) for 5 min at room temperature to remove the red blood cells.

### Cell Surface Staining, Intracellular Cytokine Staining, and Flow Cytometry

Single-cell suspensions were pretreated with anti-CD16/32 antibody (clone: 2.4G2; TONBO Biosciences, San Diego, CA, USA) to block the Fc receptors. The cells were subsequently stained with the antibody mixture of CD45.2 (clone: 104; BioLegend, San Diego, CA, USA) and CD49f (clone: GoH3; eBioscience, San Diego, CA, USA). Propidium iodide (Invitrogen™, Thermo Fisher Scientific) was used to exclude the dead cells. For intracellular cytokine staining, the cells were stained with surface markers prior to fixation and permeabilization using Cytofix/Cytoperm kit (BD Biosciences, San Jose, CA, USA), according to the manufacturer’s protocol. Anti-IL-23p19 (clone: fc23cpg; eBiosciences) and RatIgG1κ (clone: RTK2071; BioLegend) were used as isotype controls. The stained samples were examined using LSRFortessa™ Cell Analyzer (BD Biosciences). The flow cytometry data were analyzed using FlowJo software (TreeStar, Ashland, OR, USA).

### Real-Time Quantitative PCR

Total RNA from HaCaT and JurkaT cells was isolated using RNeasy Mini kit (Qiagen, Valencia, CA, USA) and then reverse-transcribed into cDNA using PrimeScript™ RT Master Mix (Takara Bio, Shiga, Japan). Real-time quantitative PCR was performed using SYBR™ Green (Takara Bio) and QuantStudio™ 3 Real-Time PCR System (Applied Biosystems, Thermo Fisher Scientific) by monitoring the synthesis of double-stranded DNA during PCR cycles. For each sample, duplicate test reactions were analyzed for the expression level of the gene of interest, with hypoxanthine guanine phosphoribosyltransferase (*Hprt*) as the internal control for normalization. The primers used were as follows: *Hprt* (Forward: 5’-CACAGGACTAGAACACCTGC-3’, Reverse: 5’-GCTGGTGAAAAGGACCTCT-3’) and *IL36G* (Forward: 5’-AGAGTAACCCCAGTCAGCGTG-3’, Reverse: 5’-AGGGTGGTGGTACAAATCCAA-3’).

### Immunohistochemical Analysis

For histological analysis, we used human skin samples from the skin biopsies of patients at the Ajou University Hospital after obtaining written informed consent (AJIRB-MED-KSP-21-376). Hematoxylin and eosin staining and immunohistochemical analysis of formalin-fixed, paraffin-embedded specimens were performed using primary antibodies against IL-36γ (R&D Systems, Minneapolis, MN, USA), IL-23 (BioLegend), IL-17A (Abcam, Cambridge, UK), and IFN-γ (Abcam). Polink-2 plus HRP detection kit for mouse and rabbit antibodies (GBI Labs, Mukilteo, WA) was used for the detection of the primary antibodies. The immunohistochemical results from epidermal samples were analyzed using Image-Pro Plus version 4.5 (Media Cybernetics Co., Silver Spring, MD, USA). The expression level (image analysis score) in each stained sample was determined by calculating the ratio of the stained area to the measured area on the representative area of each specimen.

### Statistical Analysis

The data are presented as means ± standard deviations. Statistical analyses were performed using GraphPad Prism 5 software (GraphPad Software, Inc., San Diego, CA, USA) and R package (R Foundation for Statistical Computing, Vienna, Austria). Unpaired Student’s *t*-test was used to compare the data between two groups. Differences between groups were considered significant if *P <*0.05.

## Data Availability Statement

The datasets presented in this study can be found in online repositories. The names of the repository/repositories and accession number(s) can be found in the article/**Supplementary Materials**.

## Ethics Statement

The studies involving human participants were reviewed and approved by AJIRB-MED-KSP-21-376. Ajou University Hospital. The patients/participants provided their written informed consent to participate in this study. The animal study was reviewed and approved by Young Joon Park Ajou University School of Medicine.

## Author Contributions

Conceptualization: YP, LE-S, and YCK; Methodology: YP, YHK, and YCK; Data curation: YP; Formal Analysis: YP, YHK, and YCK; Writing – original draft: YP, YHK, and YCK; Writing – review & editing: YP, LE-S, and YCK. All authors contributed to the article and approved the submitted version.

## Funding

This work was supported by the GRRC program of Gyeonggi Province [grant number GRRCAJOU2016B04]. The GRRC program of Gyeonggi Province had no involvement in the study design; collection, analysis, and interpretation of data; and writing the manuscript.

## Conflict of Interest

The authors declare that the research was conducted in the absence of any commercial or financial relationships that could be construed as a potential conflict of interest.

## Publisher’s Note

All claims expressed in this article are solely those of the authors and do not necessarily represent those of their affiliated organizations, or those of the publisher, the editors and the reviewers. Any product that may be evaluated in this article, or claim that may be made by its manufacturer, is not guaranteed or endorsed by the publisher.

## Supplementary Material

Supplementary Figure 1 Clustering and analysis of single-cell RNA sequencing (scRNA-seq) data using CellChat R package. **(A, B)** Dot plots showing the markers of each cluster in for GSE151177 **(A)** and GSE162183 **(B)**. **(C)** CellChat plot showing the number of interactions between clusters of GSE162183.

Supplementary Figure 2 Change in ear thickness and gating strategies of isolated cells from imiquimod-induced mice skin. **(A)** Representative gating strategy of CD45.2- and CD45.2+ cells from mice whole skin. **(B)** Changes in the ear thickness of the control and imiquimod-treated mice. **(C)** Representative gating strategy of cells from mice epidermis. Results are representative of two independent experiments with six mice per group. Two mice were pooled for detailed visualization of IL-23p19+ cells for each experiment (n=3).

Supplementary Figure 3 Clustering keratinocytes from single-cell RNA sequencing (scRNA-seq) data and confirmation of *IL36G* expression. **(A, B)** Dot and feature plots showing the markers on the different layers of the epidermis in GSE151177. **(C)** Feature plot showing the markers on the different layers of the epidermis in GSE162183. **(D)** UMAP of the keratinocytes and the expression levels of *KRT1* and *KRT5* by clusters. **(E)** Expression of *IL36G* in the suprabasal and basal layers of control and psoriatic skins in GSE162183. **(F)** Relative expression of *IL36G* in the HacaT and JurkaT cells after stimulation by recombinant human IL-12 and/or IL-23.

Supplementary Figure 4 IL-23 subunit p40 blockade has no significant effect on the IFN-γ or IL-17A expression in psoriatic epidermis. **(A)** IFN-γ expression on paraffin-embedded sections of biopsies obtained from psoriatic skin treated with anti-p40 monoclonal antibody (ustekinumab) (n=8) and methotrexate-treated skin (n=9). **(B)** IL-17A expression on paraffin-embedded sections of biopsies obtained from psoriatic skin treated with anti-p40 monoclonal antibody (ustekinumab) (n=8) and methotrexate-treated skin (n=9).

## Abbreviations

scRNA-seq, single-cell RNA sequencing.
