# Supplementary figures and images for: Comparative Analysis of Single-Cell Transcriptome Data Reveals a Novel Role of Keratinocyte-Derived IL-23 in Psoriasis
Source: Front Immunol. 2022 May 25;13:905239. doi: 10.3389/fimmu.2022.905239 (PMC9174585; doi:10.3389/fimmu.2022.905239)

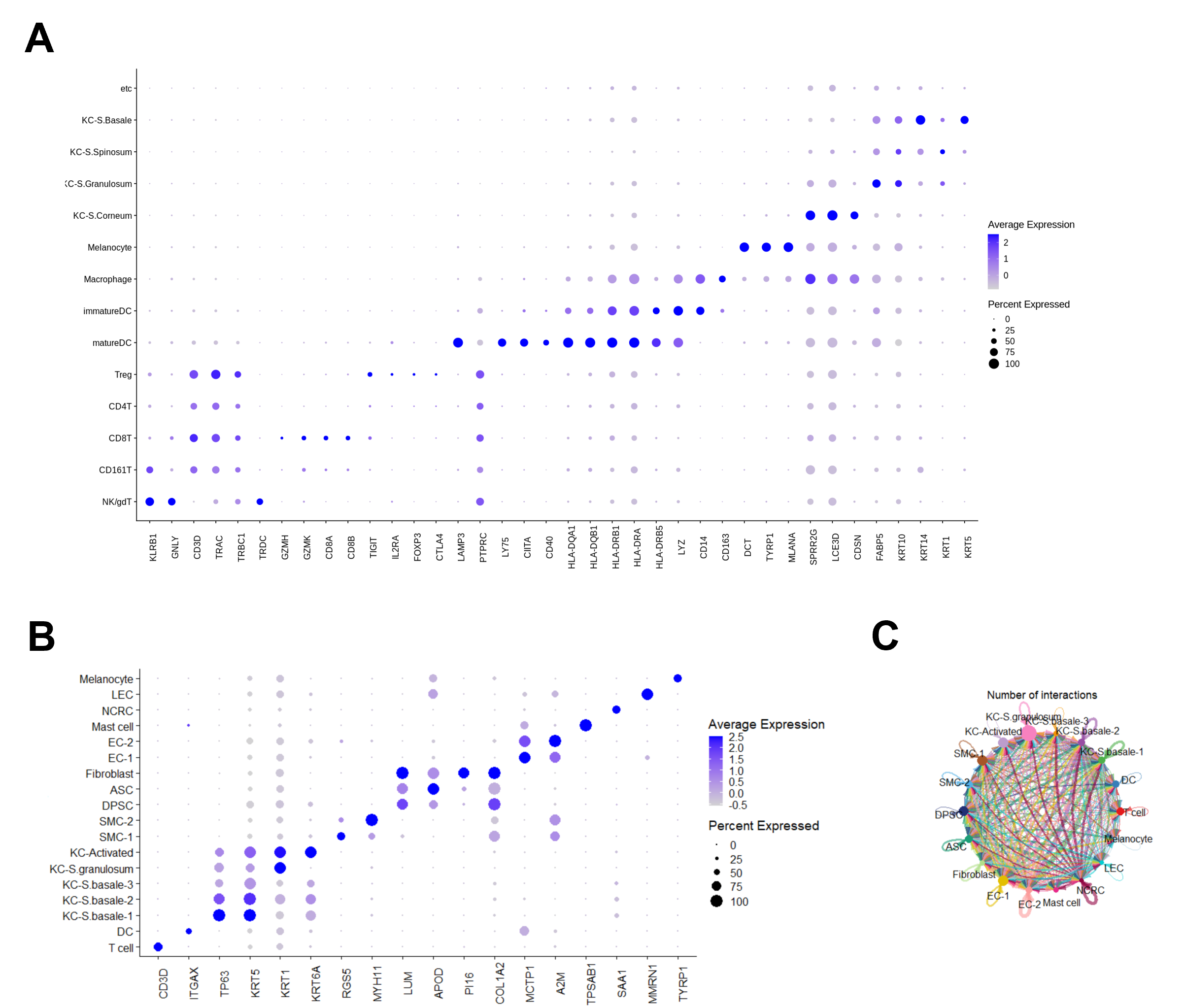

Supplement: Supplementary Figure 1 — Clustering and analysis of single-cell RNA sequencing (scRNA-seq) data using CellChat R package. (A, B) Dot plots showing the markers of each cluster in for GSE151177 (A) and GSE162183 (B). (C) CellChat plot showing the number of interactions between clusters of GSE162183. [file Image_1.tif]

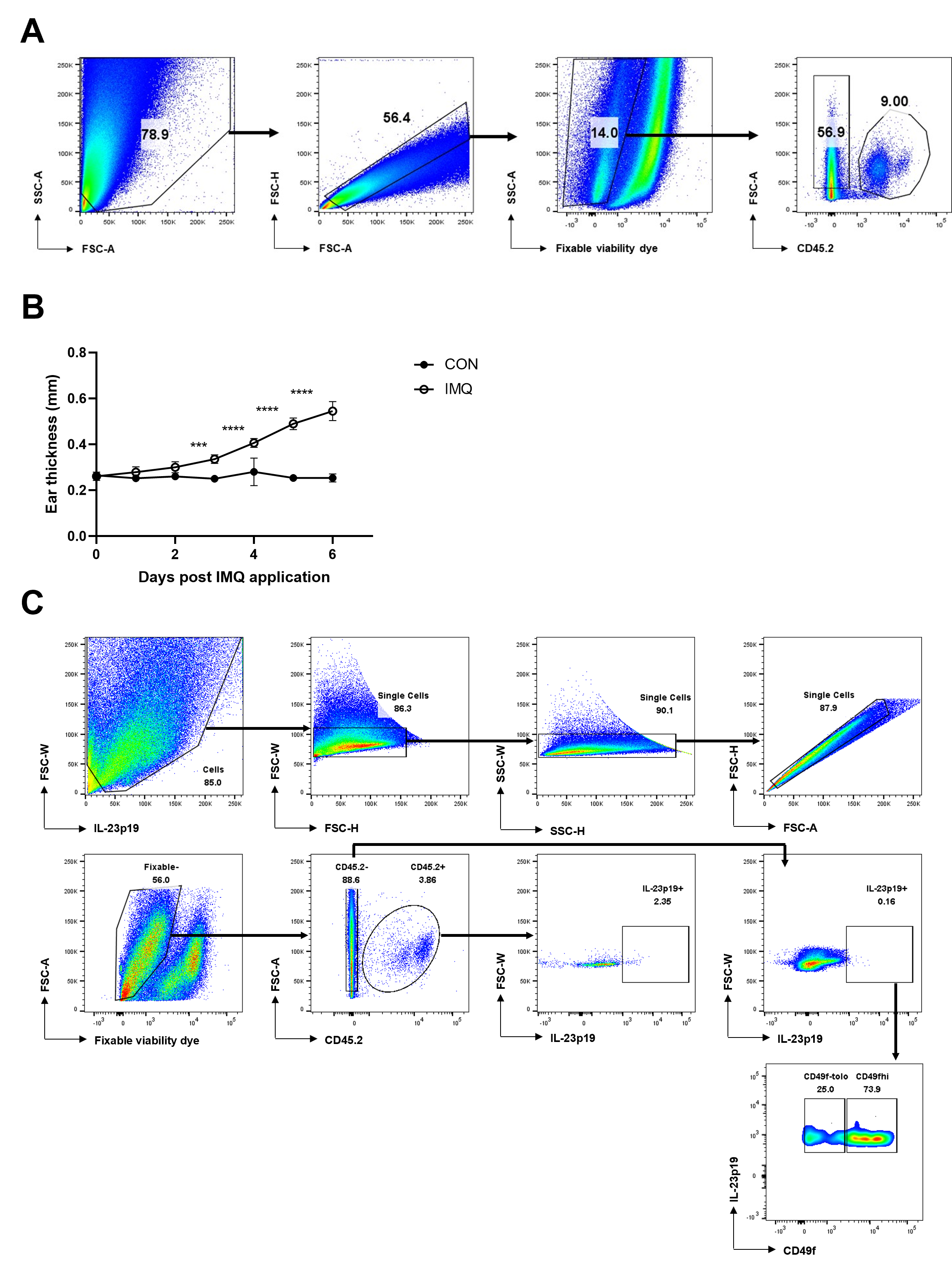

Supplement: Supplementary Figure 2 — Change in ear thickness and gating strategies of isolated cells from imiquimod-induced mice skin. (A) Representative gating strategy of CD45.2- and CD45.2+ cells from mice whole skin. (B) Changes in the ear thickness of the control and imiquimod-treated mice. (C) Representative gating strategy of cells from mice epidermis. Results are representative of two independent experiments with six mice per group. Two mice were pooled for detailed visualization of IL-23p19+ cells for each experiment (n=3). [file Image_2.tif]

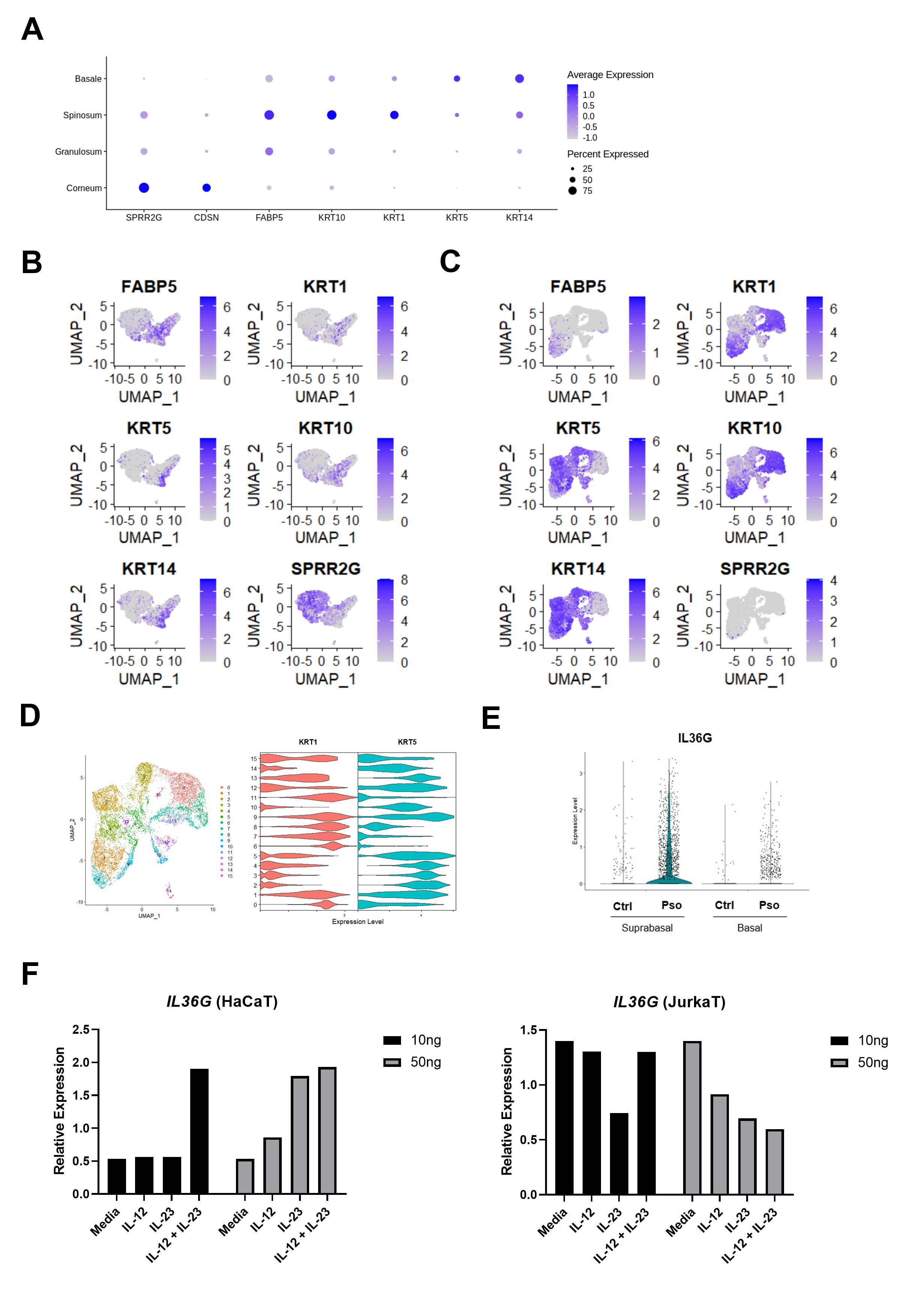

Supplement: Supplementary Figure 3 — Clustering keratinocytes from single-cell RNA sequencing (scRNA-seq) data and confirmation of IL36G expression. (A, B) Dot and feature plots showing the markers on the different layers of the epidermis in GSE151177. (C) Feature plot showing the markers on the different layers of the epidermis in GSE162183. (D) UMAP of the keratinocytes and the expression levels of KRT1 and KRT5 by clusters. (E) Expression of IL36G in the suprabasal and basal layers of control and psoriatic skins in GSE162183. (F) Relative expression of IL36G in the HacaT and JurkaT cells after stimulation by recombinant human IL-12 and/or IL-23. [file Image_3.tif]

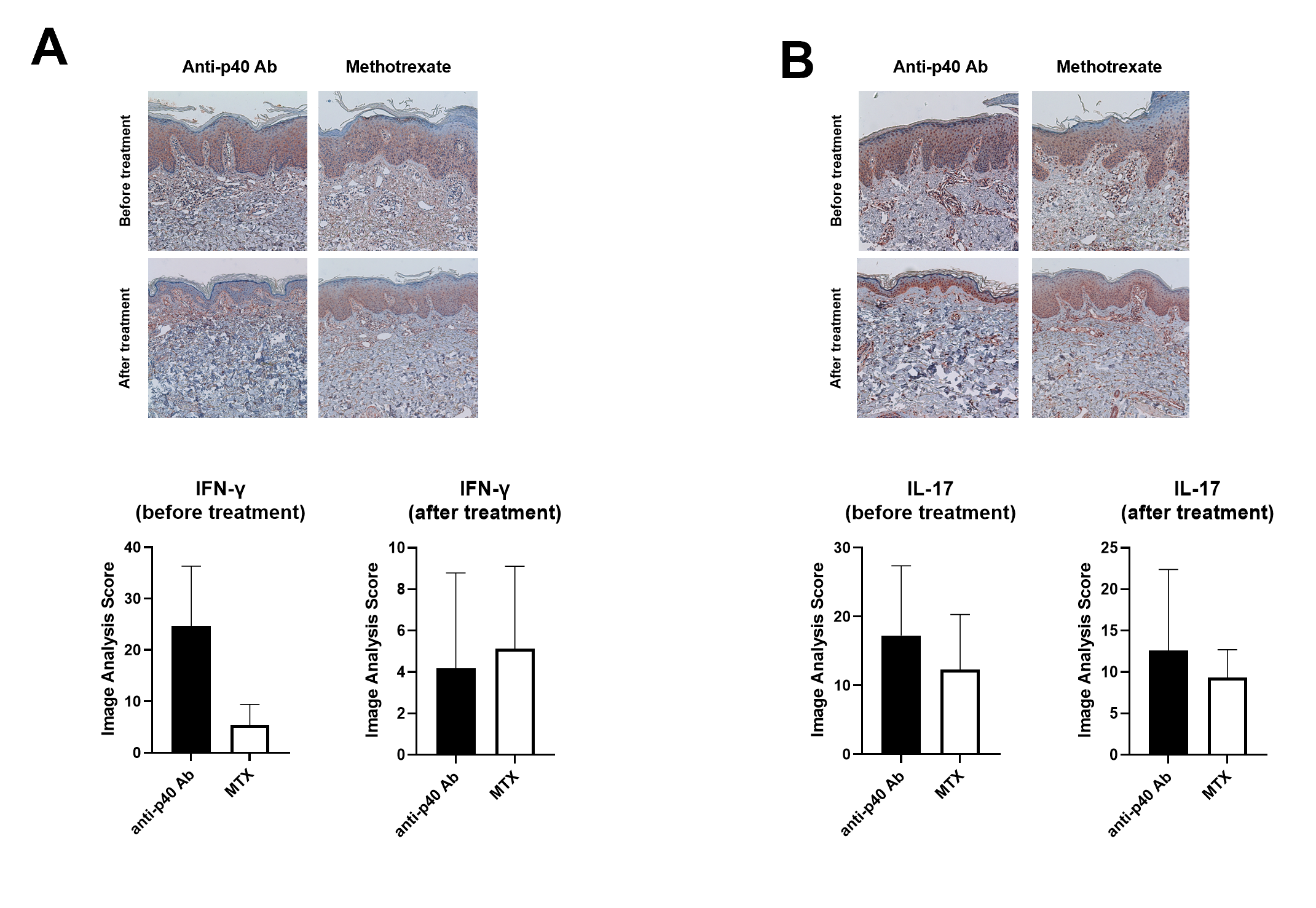

Supplement: Supplementary Figure 4 — IL-23 subunit p40 blockade has no significant effect on the IFN-γ or IL-17A expression in psoriatic epidermis. (A) IFN-γ expression on paraffin-embedded sections of biopsies obtained from psoriatic skin treated with anti-p40 monoclonal antibody (ustekinumab) (n=8) and methotrexate-treated skin (n=9). (B) IL-17A expression on paraffin-embedded sections of biopsies obtained from psoriatic skin treated with anti-p40 monoclonal antibody (ustekinumab) (n=8) and methotrexate-treated skin (n=9). [file Image_4.tif]

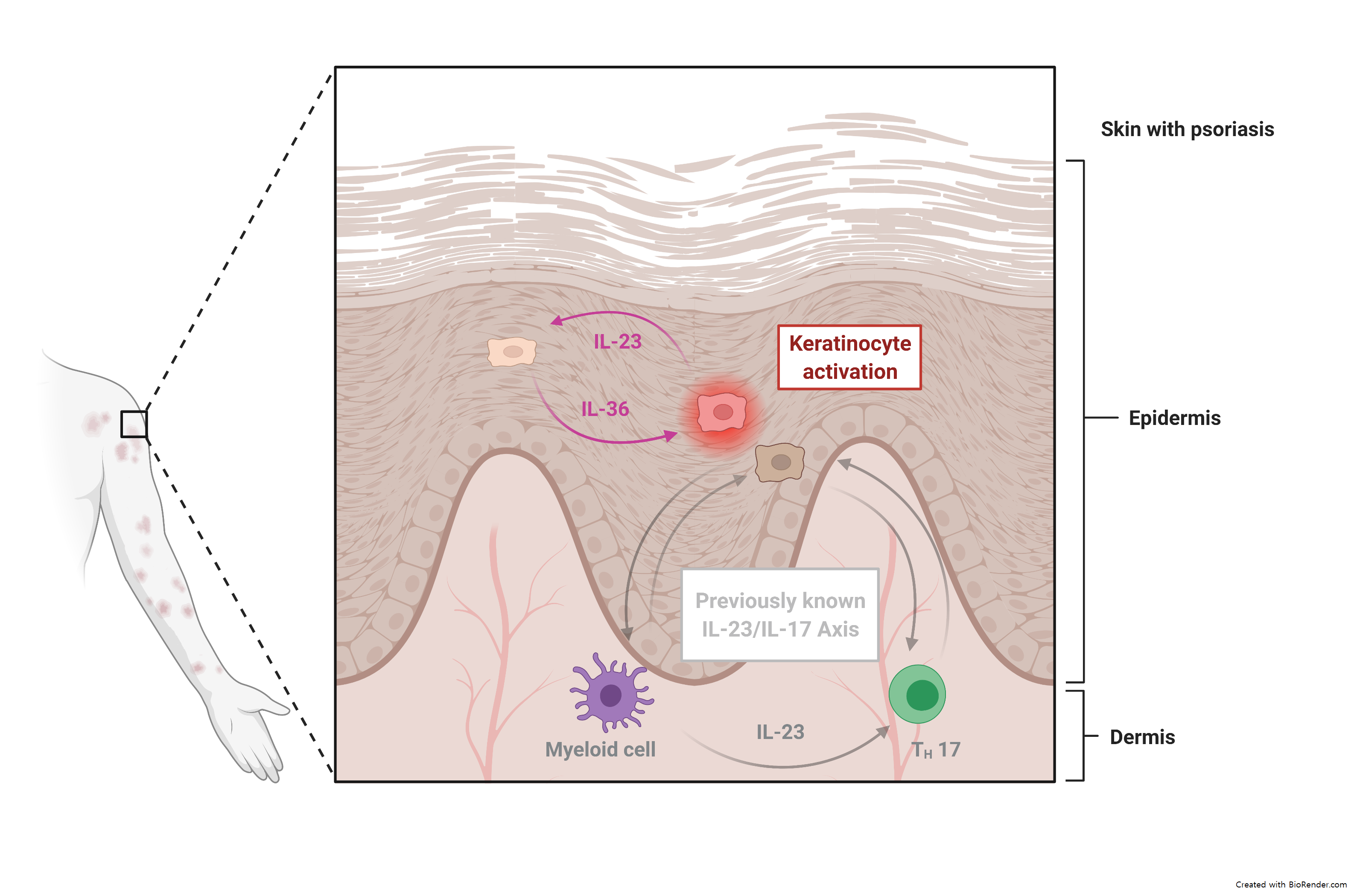

Supplement: Supplementary file 5 [file Image_5.tiff]
